# Proximal Left Main Coronary Artery Aneurysm Presenting as ST-Elevation Myocardial Infarction Treated by Stenting

**DOI:** 10.1155/2020/8833917

**Published:** 2020-11-06

**Authors:** Vijay Chander Vinod, Zuhair Eltayeb Yousif, Najat Omer Salim, Talib Majwal

**Affiliations:** Mediclinic City Hospital, Dubai, UAE

## Abstract

Coronary artery aneurysm (CAA) is a rare cardiac anomaly with a reported incidence of 0.3-4.9% of patients who undergo coronary angiography. The term is used when the coronary artery diameter exceeds more than 50% or 1.5 times the reference diameter. It can be congenital or acquired. The commonest acquired cause in an adult is atherosclerosis and in a child is Kawasaki's disease. The commonest culprit vessel is the Right Coronary Artery (RCA), followed by Left Circumflex (LCx) and Left Anterior Descending (LAD). Left main coronary aneurysms are extremely rare in clinical practice. Coronary angiography is the gold standard procedure, both for diagnosis and treatment. We report a 49-year-old male who presented with anterior wall ST-Elevation Myocardial Infarction (STEMI). The initial angiography showed LAD stent thrombosis, but when the second angiography was done, there was spontaneous recanalization of the LAD. Coronary angiography was performed at our hospital, which revealed a long left main coronary artery aneurysm measuring 9.8 mm—maximum diameter. This was treated with a size 5 × 24 mm Begraft coronary stent.

## 1. Introduction

Coronary artery aneurysm (CAA) is a rare cardiac anomaly with a reported incidence of 0.3-4.9% of patients who undergo coronary angiography. The term is used when the coronary artery diameter exceeds more than 50% or 1.5 times the reference diameter [1, 2].

It can be congenital or acquired. The commonest acquired cause in an adult is atherosclerosis and in a child is Kawasaki's disease. The commonest culprit vessel is the Right Coronary Artery (RCA), followed by Left Circumflex (LCx) and Left Anterior Descending (LAD). Left main coronary aneurysms are extremely rare in clinical practice. It is usually diagnosed incidentally either during a coronary angiogram or intravascular ultrasound or during postmortem. Coronary angiography is the gold standard procedure, both for diagnosis and treatment.

## 2. Study Setting

This case has been reported at the Emergency Department (ED) of Mediclinic City Hospital (MCH) situated in Dubai Health Care City (DHCC), Dubai, United Arab Emirates. MCH is a 230 bedded multidisciplinary tertiary care hospital and a Level 1 Cardiac Centre in the region. We see around 45,000 patients per year in our ED, of which 2400 patients present with cardiac symptoms. On average, the Cardiac Catheterization Lab carries out 300 coronary angioplasty procedures per year.

## 3. Case Presentation

A 49-year-old male was referred to our Emergency Department (ED) from a local hospital where he presented with a 10-hour history of central chest pain, radiating to the left arm and upper back. There was no history of shortness of breath, palpitations, or exertional symptoms; no history of headache, dizziness, or syncope; and no history of fever or respiratory symptoms. He has documented coronary artery disease with previous percutaneous coronary intervention to Left Anterior Descending and Ramus arteries. Other cardiovascular risk factors include hypertension, dyslipidemia, smoking, and first-degree family history of premature coronary artery disease.

**Figure 1:**
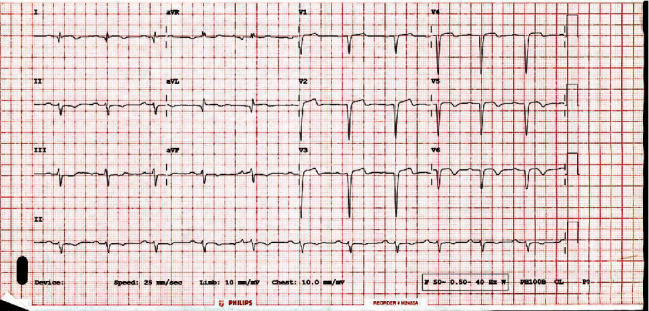
ECG at presentation.

Electrocardiogram (ECG) showed ST-segment elevation and T wave inversion in Leads I, aVL, and V1-V6. ST-segment depression in II, III, and aVF confirms acute STEMI (Figure 1).

**Figure 2:**
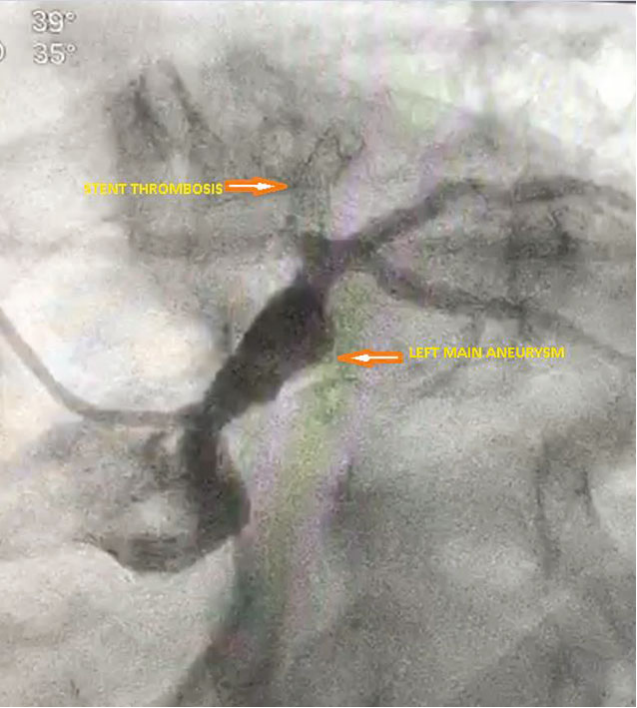
Initial coronary angiogram showing left main coronary aneurysm and stent thrombosis.

Urgent coronary angiography was performed which revealed long left main aneurysm with thrombus, stent thrombosis of LAD, patent Ramus, ectatic RCA, and severe left main ectasia (Figure 2).

At the local hospital, he had been advised to have urgent Coronary Artery Bypass Grafting (CABG). Hence, the patient came to Mediclinic City Hospital for a second opinion regarding surgical versus medical management. On admission to our hospital, the patient's pain scale was 1/10. Vitals were stable. ECG still showed the same changes as in the local hospital. Examination of the cardiovascular and respiratory systems was normal. Abdomen examination was unremarkable. Full blood count (FBC), C-reactive protein (CRP), urea and electrolytes, prothrombin time (PT), and international normalized ratio (INR) were all within normal limits. Troponin I was 0.668 ng/ml (<0.034), and creatine kinase (MB fraction) was 16.6 ng/ml (0.00-5.10). Echocardiography showed impaired left ventricular (LV) function with an ejection fraction (EF) of 35-40%. The septum and apex were akinetic. There was hypokinesia of the anterior and inferior walls. Dilated LV and LV apical clot were noted. The patient was admitted to the Intensive Care Unit (ICU) and was maintained on antiplatelet and anticoagulant therapy.

**Figure 3:**
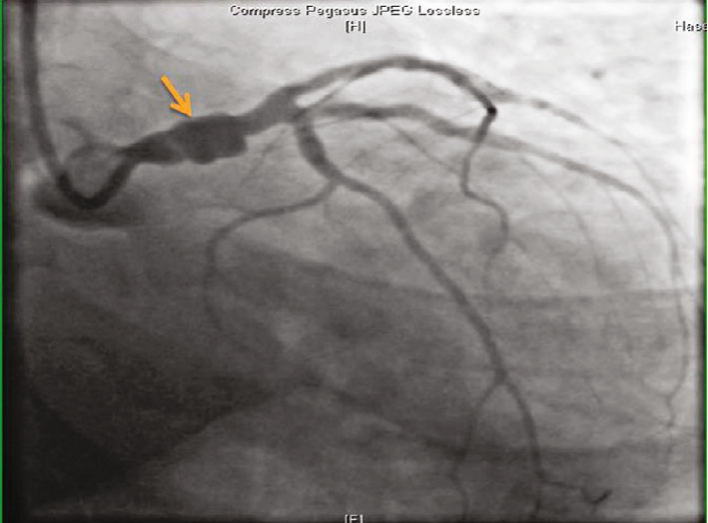
AP caudal view showing left main coronary artery aneurysm.

**Figure 4:**
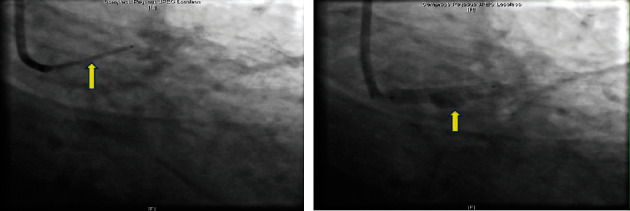
Positioning and deploying the stent in the left main coronary artery.

The revascularization strategy was discussed amongst the multidisciplinary team. It was a one-stage procedure, as the initial angiography showed LAD stent thrombosis, but when the second angiography was done, there was spontaneous recanalization of the LAD. A decision was made to go ahead with PCI, because if surgical revascularization was performed, there would be a risk of thrombus formation in the LMCA with distal embolization to the LAD/LCx resulting in STEMI, thus increasing the morbidity and mortality of the patient. Coronary angiography was performed at our hospital, which revealed a long left main coronary artery aneurysm measuring 9.8 mm—maximum diameter. This was treated with a size 5 × 24 mm Begraft coronary stent, and there was spontaneous recanalization of the LAD (Figures 3 and 4).

**Figure 5:**
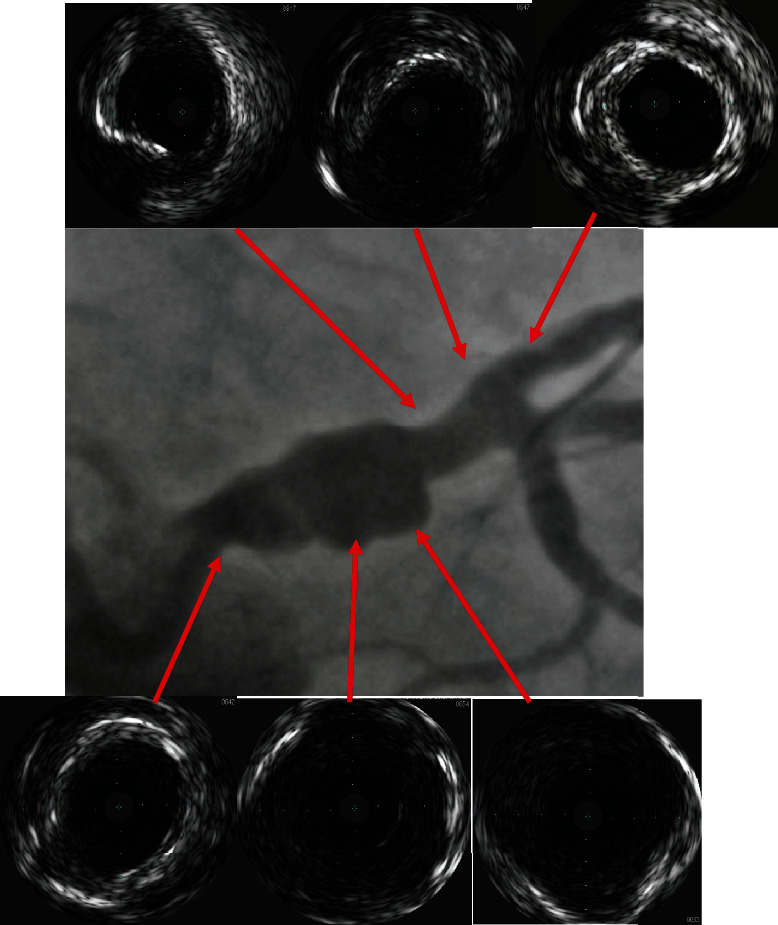
Baseline IVUS shows the aneurysm diameter and length. Healthy ostial and distal end of the left main coronary artery. Maximum diameter at aneurysm 10.6 mm, healthy segment 5.2 mm.

**Figure 6:**
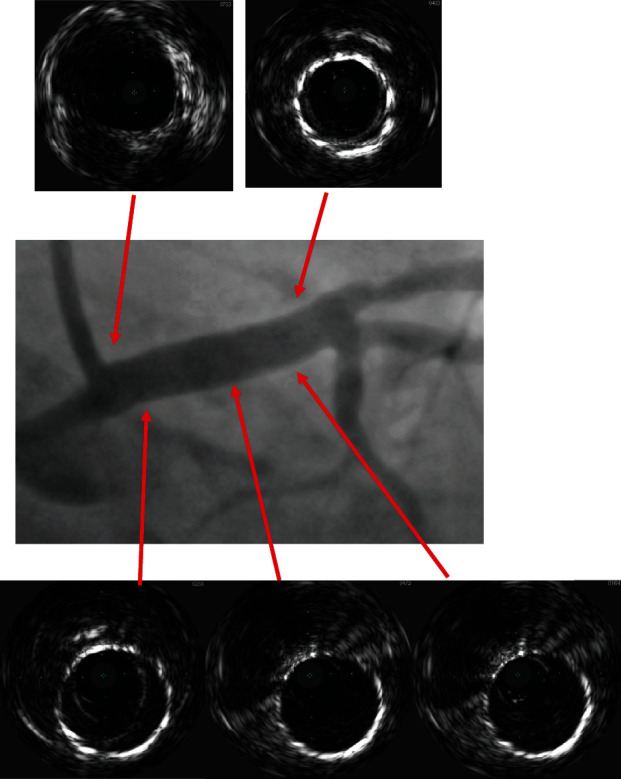
Final IVUS result with single-layer covered stent with diameter of 5 mm and complete exclusion of the aneurysm.

**Figure 7:**
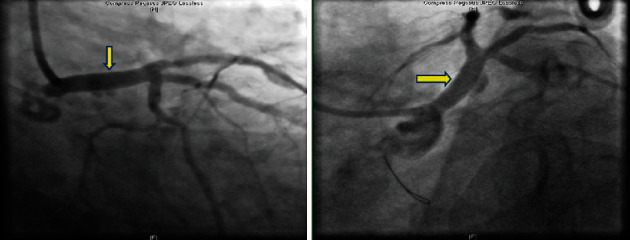
AP caudal and spider view showing post left main aneurysm stenting.

Intravascular ultrasound (IVUS) was done (Figures 5 and 6) which confirmed excellent results (Figure 7).

The patient was discharged 2 days after the procedure. He remained stable on further follow-up. Six-week control angiography was not done, and the plan is to do 1-year angiography and IVUS imaging.

## 4. Discussion

Coronary artery aneurysms (CAA) are rare in clinical practice. Left main coronary artery aneurysms (LMCAA) are extremely rare, with a reported incidence of 0.1% [3].

It can be congenital or acquired. The commonest acquired cause in an adult is atherosclerosis and in a child is Kawasaki's disease. The other causes could be connective tissue disorders like Ehlers-Danlos syndrome, Marfan syndrome, and scleroderma [4].

The commonest culprit vessel is the Right Coronary Artery (RCA), followed by Left Circumflex (LCx) and Left Anterior Descending (LAD). It is usually diagnosed incidentally either during a coronary angiogram or intravascular ultrasound or during postmortem. Coronary angiography is the gold standard procedure, both for diagnosis and treatment. Noninvasive imaging modalities such as CT coronary angiogram and MR coronary angiogram when done electively can increase the chances of detection in asymptomatic patients [5].

Various studies have reported an increased incidence of CAA after coronary angioplasty with drug-eluting stents (DES), which can range from 0.2 to 10.5%. There is an increased incidence of latent stent thrombosis in DES when compared to bare metal stents (BMS), and this is associated with increased risk of CAA [6, 7, 8]. There are case reports and literature to suggest that the chances of coronary artery aneurysms, in-stent thrombosis, and restenosis with drug-eluting stents are increasing. The preferred treatment of choice in such patients who develop coronary artery aneurysms is single-layer covered stent [9, 10, 11, 12].

The selection of coronary stents is very important, and the diameter of the coronary artery aneurysm plays an important role with regard to postprocedure complications like restenosis and in-stent thrombosis. Diameters less than 3 mm are at higher risk of restenosis, and those more than 4.5 mm have a low risk of restenosis [13]. Our patient's coronary artery aneurysm was 5 mm and has a low risk of restenosis. Moreover, the patient was started on dual antiplatelet therapy and advised to continue for life.

When it comes to treatment modality of choice, there is no statistical significance in the survival rates of patients treated either medically or surgically, between patients who had CAA and patients who had obstructive CAD but did not have CAA [1, 14].

There is still a great amount of controversy about whether to treat CAA medically or surgically. The decision is usually based on individual patient criteria like CAA with proximal stenosis or emboli from the thrombus, patient's overall clinical condition and coexisting CV risk factors, and of course the expertise of the treating physician. Surgical management is usually recommended in symptomatic patients who demonstrate thrombus within the CAA which embolised to the distal coronary segment causing myocardial ischemia. However, in our case, the LMCAA was successfully treated with stenting rather than surgical management. We deployed the new generation single-layered covered stent (polytetrafluoroethylene), which has got better safety profile, higher efficacy, and reduced risk of restenosis and in-stent thrombosis when compared to the old generation double-layered stents [15, 16].

Since these cases are extremely rare, there is no standard guideline or protocol to treat LMCAA when they present to the ED with acute myocardial infarction. Hence, this poses a diagnostic challenge to the treating clinician.

## 5. Conclusion

Coronary artery aneurysms (CAA) are rare and left main CAA is extremely rare in clinical practice. The patient we presented had left main coronary artery aneurysm (maximum diameter 9.8 mm) with a thrombus that embolised the distal coronary segment causing acute myocardial infarction. He was treated percutaneously with the deployment of 5 × 24 mm Begraft-Bentley coronary stent, with excellent angiographic and clinical outcome.
